# Editorial: Molecular insights of microbiota and innate lymphoid cell interactions

**DOI:** 10.3389/fimmu.2024.1497305

**Published:** 2024-10-11

**Authors:** José Luís Fachi, Marcela Davoli Ferreira, Niels Olsen Saraiva Câmara

**Affiliations:** ^1^ Department of Pathology and Immunology, Washington University School of Medicine, St Louis, MO, United States; ^2^ Department of Physiology and Pharmacology, Snyder Institute for Chronic Diseases, Cumming School of Medicine, University of Calgary, Calgary, AB, Canada; ^3^ Department of Immunology, Institute of Biomedical Sciences, University of São Paulo, São Paulo, Brazil

**Keywords:** innate immunity, innate lymphoid cells, mucosal barrier, signaling pathway, microbiota, metabolites, intestinal inflammation

In the dynamic field of immunology, the discovery of innate lymphoid cells (ILCs) in 2008 marked a transformative milestone, as highlighted by Vivier et al. (1). These cells, present in both humans and mice, have become central to research due to their crucial role in maintaining barrier immunity and their involvement in a range of microbiota-driven disorders.

ILCs are a diverse group of cells categorized into three main subsets based on their expression of transcription factors and cytokines (1). Group 1 ILCs (including ILC1 and NK cells) are characterized by the expression of T-bet and IFN-γ, mirroring the function of Th1 cells. Group 2 ILCs express GATA3 and cytokines such as IL-4, IL-5, and IL-13, resembling Th2 cells. Group 3 ILCs, defined by the expression of RORγt and production of IL-22 and IL-17, are analogous to Th17 cells. Although ILCs perform functions similar to adaptive T cells, they are distinguished by the absence of antigen-specific receptors. Instead, their activation and responses are mediated through cytokine and alarmin signaling.

These cells are primarily located at mucosal surfaces and other barrier sites and act as a crucial first line of defense by rapidly producing cytokines that orchestrate immune responses and maintain tissue homeostasis. Their role in health and disease is becoming increasingly recognized, particularly in relation to conditions such as inflammatory bowel diseases, cancer, and autoimmunity and metabolic disorders. A key aspect of their function is their interaction with the microbiota—a complex and diverse community of microorganisms essential for shaping host immunity. In particular, the gut microbiota influences immune cell development, modulates inflammatory responses, and impacts systemic immunity (2). Positioned strategically at these barrier sites, ILCs are well-equipped to sense microbial signals and respond accordingly, helping to maintain a balanced immune environment. Disruptions in this crosstalk can lead to dysbiosis, driving the onset and progression of various diseases. Understanding these interactions holds significant potential for developing microbiota-targeted therapies that could restore immune balance and offer new approaches to disease treatment and prevention.

One notable study in this Research Topic (3) examines the communication between the gut microbiota and ILCs, highlighting how microbial metabolites and other factors influence ILC function and thereby shape intestinal immunity. This review by Guo et al. offers promising avenues for therapeutic intervention through microbiota-targeted therapies. Similarly, another significant contribution focuses on the role of Group 3 ILCs in intestinal diseases. Li et al. highlight the specific functions of these cells within the intestine, providing insights into their role in maintaining intestinal homeostasis and their involvement in inflammatory conditions.

In addition to exploring these biological interactions, the integration of multi-omics technologies and artificial intelligence (AI) has opened new avenues for investigating the complex relationships between immune cells and their environment. The contribution from Xu et al. within this Research Topic utilizes these advanced approaches to uncover new neutrophil clusters and potential biomarkers in sepsis, offering valuable insights into the immune landscape during severe infection. This research not only enhances our understanding of sepsis but also illustrates the potential of integrating multi-omics and AI in studying innate immune cells and microbiota interactions. Moreover, Huang et al. feature a comprehensive bibliometric and visual analysis of global research trends on ILCs in the brain, gut, and lung, highlighting the emerging focus on the brain-gut-lung axis. By analyzing over 8,000 articles, the study identifies key contributors, influential publications, and evolving research hotspots, such as the growing interest in tumor immunity and the tumor microenvironment. The analysis reveals that while the United States leads in publications and citations, China is becoming a significant player, though with less global impact. The study concludes that the brain-gut-lung axis and its regulation by ILCs remain underexplored, signaling a crucial area for future research.

Overall, the studies presented in this Research Topic emphasize the fundamental role of ILCs in maintaining health and their potential as therapeutic targets in various diseases. As our understanding of ILC biology continues to advance, it is becoming increasingly clear that these cells are central to mediating interactions between the microbiota and the immune system. The integration of advanced technologies, such as multi-omics and AI, is poised to accelerate discoveries in this field, offering new insights into the molecular mechanisms regulating ILC function and their interactions with the microbiota.

For a better understanding of ILCs, it is essential to investigate their interactions with the microbiome more thoroughly, particularly across different tissues and disease contexts. Such research could pave the way for innovative therapeutic strategies that address the complex relationships between ILCs and the microbiota, potentially offering new treatments for a variety of conditions. This Research Topic represents a significant advancement in this effort, and we encourage continued exploration in this area, looking forward to further growth and impact in the field.
